# Functional Recovery After Hip Arthroplasty with a Minimal Invasive or Classical Approach Eight Years After Intervention

**DOI:** 10.3390/jfmk9040208

**Published:** 2024-10-26

**Authors:** Mirela Vuckovic, Lana Ruzic, Karlo Tudor, Tomislav Prpic, Zdravko Jotanovic, Silvije Segulja, Andrica Lekic, Ksenija Bazdaric

**Affiliations:** 1 Department of Physiotherapy, Faculty of Health Studies, University of Rijeka, Viktora Cara Emina 5, 51 000 Rijeka, Croatia; 2 Department of Sport and Exercise Medicine, Faculty of Kinesiology, University of Zagreb, 10000 Zagreb, Croatia; lana.ruzic.svegl@kif.unizg.hr; 3 University Hospital for Orthopaedics and Traumatology Lovran, Faculty of Medicine, University of Rijeka, Viktora Cara Emina 5, 51000 Rijeka, Croatia; karlotudor@yahoo.com (K.T.); tomislav.prpic@uniri.hr (T.P.); zdravko.jotanovic@uniri.hr (Z.J.); 4 Faculty of Health Studies, University of Rijeka, Viktora Cara Emina 5, 51000 Rijeka, Croatia; 5 Department of Clinical Medical Sciences I, Faculty of Health Studies, University of Rijeka, Viktora Cara Emina 5, 51000 Rijeka, Croatia; silvije.segulja@fzsri.uniri.hr; 6 Department of Basic Medical Sciences, Faculty of Health Studies, University of Rijeka, Viktora Cara Emina 5, 51000 Rijeka, Croatia; andrica.lekic@uniri.hr (A.L.); ksenija.bazdaric@uniri.hr (K.B.)

**Keywords:** arthroplasty, replacement, hip, functional status, minimally invasive surgical procedures, treatment outcome

## Abstract

**Background:** The aim of this study was to investigate differences in functional recovery eight years after total hip arthroplasty in patients who underwent hip joint surgery using two different approaches: the classic lateral approach and the anterolateral minimally invasive surgical approach. **Methods:** Eight years after the hip replacement, 68 subjects, 32 in the classic and 36 in the minimally invasive group, underwent follow-up measurements involving the Harris Hip Score (HHS), range of motion, strength of the abductor muscles, 50-m walk time, body mass index (BMI), physical activity questionnaire, and visual analogue scale (VAS) pain during general activities. **Results:** Higher HHS (*p* < 0.001), hip abduction (*p* < 0.001), and hip flexion (*p* = 0.018) range of motion values were obtained in the minimally invasive approach group. A correlation between physical activity (PA) and the hip abduction muscle strength in the classic group (r = 0.43; *p* = 0.011) and a correlation between PA and the HHS in the minimally invasive group (r = 0.34, *p* = 0.041) was found. BMI was correlated with the 50-m walk time in both groups (classical: r = 0.39; *p* = 0.027; minimally invasive r = 0.35; *p* = 0.030); meanwhile, in the minimally invasive group, BMI was negatively correlated with hip flexion (r = −0.37; *p* = 0.020). **Conclusions:** Eight years after total hip arthroplasty, performed using either an anterolateral minimally invasive or lateral approach, there was no difference in the patients’ functional outcome in relation to BMI. The minimally invasive approach benefits patients by granting them better functional abilities. A clinical difference was found in the HHS, in favour of the minimally invasive group.

## 1. Introduction

Types of arthroplasty and surgical techniques are constantly evolving and there are conflicting opinions regarding which surgical approach is preferable for hip replacement. The development of surgical techniques with appropriate physiotherapeutic interventions should improve patients’ functional outcomes in the long term. Minimally invasive surgery (MIS) is interpreted and described very differently in the literature; therefore, the results of scientific research also vary. For some, it is only a small surgical incision, while other authors spare the surrounding muscles and make a small surgical incision. Tudor et al. outlined the importance of sparing the abductor muscles of the hip joint during surgery and recommended that this be used to define MIS [1]. Despite various studies, the scientific community is divided regarding the superiority of the MIS compared to the classical lateral surgical approach (i.e., classical surgery—CS) [2,3,4,5]. The advantages of MIS are that it is associated with less blood loss during surgery, less postoperative pain, a shorter hospital stay, a shorter recovery time, and a smaller surgical incision [6,7]. On the other hand, other authors report a greater number of complications in MIS, with an increased risk of neurovascular damage and a higher prevalence of dislocations [5,8].

The surgeon’s typical recommendations, based on experience and the longevity of the artificial joint, include physical exercise and low-intensity activities such as swimming, riding a stationary bike, dancing, bowling, and walking [9]. A large number of studies associate a high body mass index (BMI) (>30) with poorer early and mid-operative outcomes, including longer hospital stays, the prolonged use of analgesics in the early phase, the extended use of assistive devices [10,11,12], an increased risk of bleeding, infection, dislocation [13,14,15], and a higher mortality rate [16]. There are also studies claiming the opposite [17,18], but only a few studies have investigated the long-term effects of BMI on the functional outcomes of the patient [1,19,20].

The early postoperative outcomes of both MIS and CS have been extensively studied [21,22,23], but there is a significant lack of more extensive clinical, prospective, and longitudinal studies; we therefore felt the need to determine whether the superiority of a particular approach persists after many years. In addition, there are conflicting opinions about the level of physical activity (PA) that can be undertaken after total hip arthroplasty (THA) and about the relationship between BMI and the outcomes of surgery, which is not supported by the existing rehabilitation paradigm. To date, it is not clear which surgical approach is superior, MIS or CS.

Therefore, the aim of this study was to determine the differences in functional recovery, eight years after THA, between a group of patients operated on using the classic lateral approach (CS) and a group operated on using the minimally invasive anterolateral approach in the hip joint (MIS). We also examined the role of PA and BMI as mediators in this process.

## 2. Materials and Methods

### 2.1. Participants

**Figure 1:**
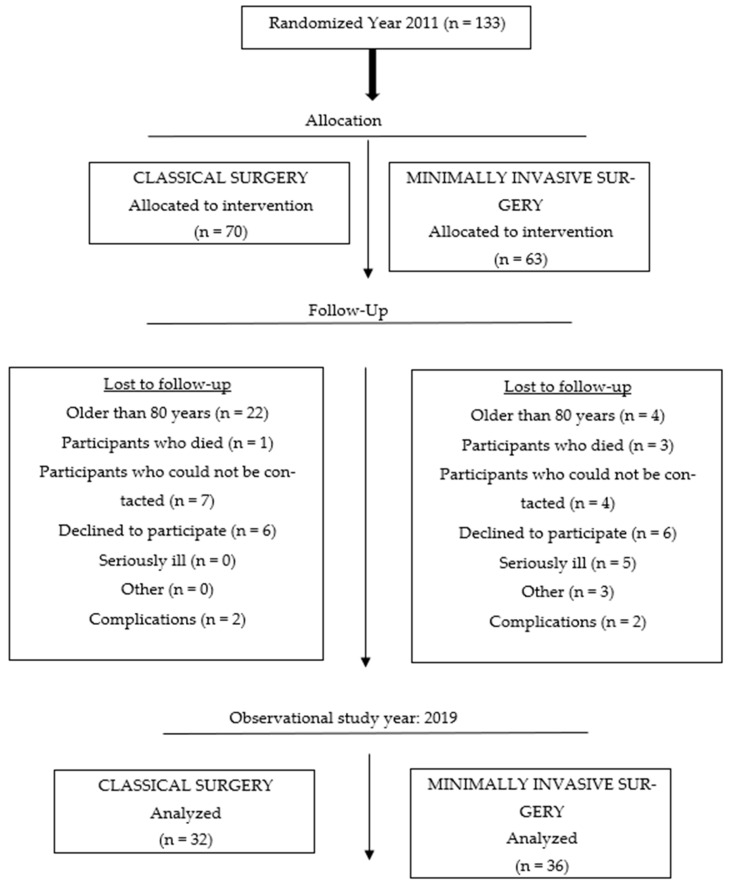
Flowchart of the number of participants at different stages in the study.

This study was conducted in 2019 at the University Hospital for Orthopaedics and Traumatology Lovran, School of Medicine, University of Rijeka, Croatia, and lasted from April to October. This part of the study was transversal, but it was also the third and final part of a prospective longitudinal study [1] that began in 2011 and whose original sample comprised 133 respondents divided into two groups (70 in the CS group and 63 respondents in the MIS group) (Figure 1). The subjects in the sample underwent an elective surgical procedure that involved the insertion of an artificial hip joint in 2011. The inclusion criterion was primary osteoarthritis of the hip joint, while the exclusion criterion was hip dysplasia. The stratified randomization method was employed to ensure that there was balance among the groups regarding age and gender. After identifying and assigning all the subjects into blocks, simple randomization was applied [1].

Once the data had been processed, 68 patients were included in the study, i.e., 32 in the CS group and 36 in the MIS group, with an equal response rate in both groups.

A total of 104 patients were contacted, which corresponds to a response rate of 65% (patients who were > =80 years old in 2011 were not included in the call).

To determine the sample size, we used two methods: I. an a priori analysis estimated that a minimum of 52 subjects were required to perform two repeated measures in two groups to achieve 80% power with a small-to-medium effect factor (f = 0.20) and a significance level of alpha 0.05, and II. the number of subjects in similar studies was estimated [24,25], but a shorter period was used to monitor the outcome.

### 2.2. Surgical Technique

The classic lateral approach to total hip arthroplasty is a technique consisting of a longitudinal incision and the partial dissection of the gluteus medius and gluteus minimus muscles (Bauer/Hardinge technique). An anterolateral minimally invasive surgical approach based on the classic anterolateral approach was then developed (Watson-Jones). The main feature of this approach is that the hip joint is accessed between the gluteus medius muscle and the tensor fasciae latae muscle without separating the muscle attachments. All operations were performed by two high-volume surgeons. One operated on patients using the classic approach, while the other used the minimally invasive approach. Standard techniques and instruments were employed for both approaches, depending on the procedure.

### 2.3. Study Design

The STROBE Statement checklist [26] was applied in this study. All the measurements were performed by the same physiotherapist (investigator). This part of the study was transversal, but it was also the final part of a prospective longitudinal study. Patients came for regular check-ups at 6 weeks, 3 months, 1 year, and 3 years after the surgical procedure. All motor skills were recorded. After these follow-ups, patients were no longer invited for follow-up because we wanted to observe the motor skills in both groups after a longer period of time. Due to the age of the participants, we decided to invite them for follow-up again after 8 years. The study consisted of three phases. In the first phase of the study, the patients were invited for a follow-up examination with an orthopedist and for tests with a physiotherapist via telephone or e-mail. The patients received the exact date and time of their appointment. The second and third phases took place on the same day. In the second phase, the patient underwent a follow-up examination with the orthopedist who performed the surgery. In the third phase, which followed the examination, the physiotherapist tested the patients’ motor skills and administered questionnaires. The questionnaires were completed under the supervision of the researcher to avoid the patients making any mistakes during the completion process. The physiotherapist also conducted the tests in the clinic, except for the walking test, which was performed in the corridor. For this part, the following data were collected: morphological measurements (body mass and height), from which the BMI was calculated; a detailed medical history of existing diseases to ensure that other diseases did not influence the results of the study; the patients’ hip range of motion, abductor strength, 50 m walking distance, and pain intensity (during general activities); the International Physical Activity Questionnaire (IPAQ); and the Harris Hip Score (HHS) questionnaire.

### 2.4. Outcome Measures

#### 2.4.1. Questionnaires

The International Physical Activity Questionnaire-Short Form (IPAQ-SF) is one of the most commonly used PA questionnaires. It has been used in different countries around the world and has shown a high reliability coefficient when measuring the level of PA in numerous international studies [27,28,29]. The questionnaire can be used to categorize subjects according to their PA level into three possible categories: insufficiently physically active (0–600 MET/min/week), minimally physically active (601–3000 MET/min/week), and sufficiently physically active (>3001 MET/min/week) [30]. The total level of PA was calculated by adding the results from the above-mentioned areas.

HHS was developed to assess the success of hip joint surgery, the damage caused to the joint, and the outcome of the treatment of hip joint disease [31] by a clinician [32]. There are 10 questions covering four areas: pain, function, the absence of deformity, and the range of motion. The number of points that can be achieved ranges from 0 to 100, and the higher the number of points, the better the functional outcome. The results can be interpreted as follows: <70—poor result; 70–80—fair; 80–90—good; and 90–100—excellent [33].

#### 2.4.2. Motor Function Tests

The range of flexion and abduction movements of the hip joint were measured using a goniometer. When measuring the flexion and abduction of the hip joint, the subject lay on their back and the examiner fixed their pelvis to rule out possible compensation mechanisms.

The strength of the abduction muscles of the hip joint was measured using a hand-held dynamometer. The patient lay on their back and the tested leg was positioned against a wall. The dynamometer was attached to the wall and the subject pushed the pad of the dynamometer with the greatest possible force. Care was taken to ensure that all compensatory mechanisms were inhibited during the movement. The measurement was carried out three times and the average value was given in Newtons (N).

A 50-m walking distance was used to assess general mobility and the function of the musculoskeletal system under specific walking conditions. The start and end of the 50-m corridor were marked with coloured tape. A stopwatch was used to measure the time (in seconds) it took the subject to cross the length of the 50-m corridor.

#### 2.4.3. Pain

Pain during general activities was assessed using a one-dimensional, visual analogue scale (VAS), which is simple and easy to use. It consists of a solid line numbered from 0 to 10, with 0 (on the left side of the line) indicating the least severe pain and 10 (on the right side of the line) indicating the most severe pain. It has been shown to be reliable and valid, provided we know the target population that we are assessing [34].

#### 2.4.4. Statistical Data Processing

Descriptive and analytical methods were used to process the data. All variables were grouped as categorical and continuous values. The normality of the distribution was tested using the Kolmogorov–Smirnov test, and an appropriate method of analysis was implemented in accordance with the results obtained. The non-parametric Mann–Whitney U test for independent samples was used, and Spearman’s R coefficient was used to calculate the relationship between PA and motor functions, and between BMI and motor functions, and to test the relationship between age and motor skills. The chi-square test was used to analyze the rate of comorbidities between the groups. The power analysis for the Mann–Whitney test was performed using the online calculator Psychometrica.de (https://www.psychometrica.de/effect_size.html) [35].

The level of statistical significance in the study was determined at the level of *p* < 0.05. The data were processed using the statistical data processing programme STATISTICA version 13.5.0.17, 1984–2018 TIBCO Software Inc. Santa Clara, US, licenced for the Faculty of Kinesiology, University of Zagreb.

## 3. Results

### 3.1. Demographic Characteristics

The demographic characteristics of the subjects are described in Table 1. A total of 68 subjects participated in the study; of these, 32 were in the group operated on using CS and 36 were in the group operated on using the MIS procedure. The groups did not differ in terms of age (*p* = 0.26), gender (*p* = 0.17), or body mass index (*p* = 0.64).

The subjects did not suffer from diseases that could influence the functional results of the monitored variables. Of the non-communicable chronic diseases, the most prevalent (Table 1) were those of the cardiovascular system, which are also the main cause of mortality worldwide. The chi-square test did not show a statistically significant difference in the rate of comorbidities between the groups (*p* = 0.748).

### 3.2. Harris Hip Score and Motor Function Tests

The motor function and functional ability tests measured using HHS were compared between the CS and MIS groups (Table 2).

It was found that there was a statistically significant difference between the groups in terms of the functional outcome of the surgery, as measured by HHS (*p* < 0.001) and the range of motion in terms of hip abduction (*p* < 0.001) and hip flexion (*p* = 0.018); these results were in favour of the MIS group, with a moderate-to-strong effect size (d). The effect size for HHS was 105 (Table 2), which is a strong effect. If we take a closer look at the median of the tested variables, we can see that the values are better for all the variables tested, except for the 50 m walk and the IPAQ.

### 3.3. Relationship Between the Level of Physical Activity and Motor Functions

The relationship between the overall level of physical activity and strength in the CS and MIS groups is presented in Table 3. No difference was found between IPAQ and motor functions, except that there appears to be a statistically significant relationship between a better overall HHS and physical activity.

### 3.4. Correlation Between Body Mass Index and Motor Skills

BMI was positively correlated with the time taken to complete the 50 m walking test in both groups (Table 4). In patients with a higher BMI, the walk time was longer; meanwhile, in the MIS group, BMI was negatively correlated with hip flexion, so that a higher BMI impairs the hip flexion range of motion.

### 3.5. The Relationship Between Age and Motor Skills

In the MIS group, a correlation was found between age and the Harris Hip Score, walking 50 m, and the abductor muscle strength (Table 5). No correlation was found between age and the patients’ range of motion in terms of hip flexion, hip abduction, and level of physical activity. In the CS group, there was no correlation in any area.

## 4. Discussion

The main results of this study show that eight years after the surgery, there was a significant difference between the groups with regard to the Harris Hip Score, hip abduction, and hip flexion range of motion; these results were in favour of the MIS group. A significant correlation between physical activity and the hip abductor strength in the CS group and a positive correlation between physical activity and the Harris Hip Score in the MIS group were also found. BMI was positively correlated with the 50-m walk in both groups. There was a correlation between age and motor skills in the Harris Hip Score and hip abductor strength and the 50-m walk in the MIS group.

The association between gender and osteoarthritis (OA) in this study, regardless of the group, is consistent with epidemiological studies and the available scientific literature [36]; there are more women than men. One of the strongest predictors of hip OA is age [37,38]. This study is consistent with the literature stating that THA is most commonly performed between the ages of 60 and 80 [39].

### 4.1. Difference Between Groups in Motor Function Tests and Harris Hip Score

A difference was found between groups with regard to the patient’s range of motion in hip abduction (*p* < 0.001), hip flexion (*p* = 0.018) and Harris Hip Score (*p* < 0.001); these results were in favour of patients operated on using the MIS approach. HHS combines several functional abilities, namely, hip ROM, pain, function, and the absence of deformity. A statistically significant difference was found between the groups in HHS, with a large effect size and a clinically important difference in favour of the MIS group (excellent score) compared to the CS group (good score).

Research by Rottkay et al. on two groups (patients operated on using the MIS approach and a healthy population) showed no difference between the groups when evaluating their activities of daily living and functional abilities using HHS 12 months after surgery [40]. In a complex longitudinal follow-up study of two groups of subjects three years after THA, Tudor et al. found that most motor outcomes were in favour of the MIS group [1]. Considering that THA is mostly performed in elderly patients, it is important for patients to maintain their independence and autonomy over a long period of time, as this indirectly enhances the patients’ quality and length of life.

### 4.2. Correlation Between the Level of Physical Activity and Motor Skills

There was a correlation between physical activity and the hip abductor strength in the CS group and a correlation between physical activity and HHS in the MIS group (Table 3). Considering the surgical technique and the damage caused to the muscles during the procedure itself, it is important to note that a higher level of physical activity affects the strength of the hip abductor muscles in the CS group. In the rehabilitation process, it is important to insist that a higher level of activity (depending on the clinical picture) is performed to ensure that the patients’ neuromuscular control returns to normal as soon as possible. The strength of the hip abductor muscles not only leads to pelvic stability, but a stable pelvis also contributes to normal gait biomechanics and better mobility in the hip joint. Rosenlud et al. conducted a similar study to compare lateral and posterior approaches to hip joint arthroplasty; they found that patients operated on using a lateral approach were still limping one year after surgery [19]. This is precisely why the results achieved are important, as they equalize the functional outcomes as quickly as possible using sustained targeted exercises and enable us to assess the approach for which we expect major functional deficits. The respondents with a higher level of PA exhibited a better result in HHS, which is a functional questionnaire and usually combines all the tested variables.

### 4.3. Correlation Between BMI and Motor Skills

Surprisingly, the correlation coefficients of BMI and the functional recovery variables were low and not statistically significant in most areas, although the index was elevated in both groups; therefore, the subjects in both groups belonged to the overweight category (Table 1). A higher BMI is generally associated with lower mobility, a lower level of physical activity, and the occurrence of comorbidities and complications associated with THA [10,14,15]. There are conflicting opinions in the literature as to whether BMI has an impact on functional outcome after THA. Some authors associate BMI with a poorer functional outcome [16,17], while others state that BMI has no effect on the outcome of surgery [20,41]. Studies monitoring the long-term outcomes of surgery in obese patients found that complications and mortality were more prevalent [20,42]. However, it has been proven with certainty that patients with a higher BMI also show an improvement in the functional outcome after surgery, in comparison to the functional outcomes predicted during preoperative tests [43,44], regardless of the surgical technique employed. A review study by Courtina et al. on the influence of BMI on the functional outcome after hip and knee arthroplasty showed that there was no difference in the functional outcome [45]. In our study, an increased BMI did not influence the functional abilities of the subjects.

Because there are few studies in the literature that observe functional outcomes over a long period of time (i.e., long-term follow-up), this study provides a sound basis for future research.

The main limitations of this study are that the respondents could leave the study at any time and that it was difficult to predict the final number of participants at the beginning of the study. When a long period of time passes, it is difficult to recruit subjects and motivate them to take part in the study again. In addition, selection bias may have had an influence on the results of this study due to the loss of patients to follow-up. Furthermore, the characteristics of the patients who did not complete follow-up may have differed significantly from those who did complete the study. Finally, the difference of 4 points in HHS between groups is statistically significant and with large effect but may not be enough for a minimal clinical important difference.

## 5. Conclusions

Eight years after total hip arthroplasty using either an anterolateral minimally invasive or lateral approach, there was no difference in the patients’ functional outcome in relation to BMI.

However, the functional outcomes for patients in the MIS and CS groups were statistically different, and the MIS approach benefits patients by providing improved functional abilities. A statistical difference was found in HHS, with the MIS group exhibiting an excellent score and the CS group showing a good score.

## Figures and Tables

**Table 1:** The demographic characteristics of the subjects.

Variables	CS M (SD) or n (%)	MIS M (SD) or n (%)
Age	68.25 (8.06)	69.94 (7.3)
Gender	n (%)	n (%)
Female	24 (75)	26 (72)
Male	8 (25)	10 (28)
Body mass index	M (SD)	M (SD)
Female	27.16 (4.38)	29.11 (4.95)
Male	28 (4.69)	28.9 (3.44)
Clinical status	n (%)	n (%)
Healthy	12 (37.5)	9 (25)
Cardiovascular diseases	7 (22)	10 (27.77)
Musculoskeletal disease	1 (3.1)	0
Endocrine, nutritional, and metabolic diseases	2 (6.25)	1 (2.77)
Diseases of the respiratory system	1 (3.1)	1 (2.77)
Diseases of the genital and urinary system	1 (3.1)	0
Neoplasms	1 (3.1)	1 (2.77)
Eye diseases	0	1 (2.77)
Diseases of the digestive system	0	1 (2.77)
Two comorbidities	6 (18.75)	11 (30.55)
Three comorbidities	1 (3.1)	1 (2.77)
Operated side	n (%)	n (%)
Left side	17 (53)	17 (47)
Right side	15 (47)	19 (53)

Legend: CS—classical surgery; MIS—minimally invasive surgery; M—mean;n—number; SD—standard deviation.

**Table 2:** Differences in the motor function and Harris Hip Score between the groups.

Variables	CS C (25–75 Percentile) N = 32	MIS C (25–75 Percentile) N = 36	U *p* *	Effect Size *η*^2^ d
Abductor muscle strength	7.0 (3.0–9.0)	8.0 (5.0–9.0)	475.00 0.210	0.02 0.30
Harris Hip Score	86.3 (72.1–91.5)	92.9 (90.05–95.75)	264.00 <0.001 *	0.22 1.05
ROM-hip abduction	10.0 (10.0–15.0)	15.0 (10.0–20.0)	318.00 <0.001	0.15 0.83
ROM-hip flexion	90.0 (75.0–100.0)	95.0 (90.0–101.0)	384.00 0.018 *	0.08 0.60
Walk for 50 m	26.8 (23–32.6)	23 (20.7–31.6)	435.00 0.080	0.04 0.43
IPAQ	1389.0 (905.5–2006.0)	1265.0 (672.0–1977.0)	547.50 0.730	0.002 0.008

Legend: CS—classical surgery; MIS—minimally invasive surgery; C—median; N—number; *p*—level of statistical significance; ROM—range of movement; IPAQ—International Physical Activity Questionnaire; U—Mann–Whitney U, * statistical significance; η^2^—eta squared, d Cohen’s d.

**Table 3:** Correlation between physical activity (IPAQ) and motor functions.

Correlation of IPAQ with	CS (n = 32) r_s_ *p*	MIS (n = 36) r_s_ *p*
Abductor muscle strength	0.43 0.011 *	0.23 0.170
Walk for 50 m	−0.16 0.368	−0.33 0.052
Harris Hip Score	0.27 0.128	0.34 0.041 *
ROM-hip abduction	0.05 0.762	0.03 0.878
ROM-hip flexion	0.31 0.082	0.13 0.439

Legend: CS—classical surgery; MIS—minimally invasive surgery; r_s_—Spearman’s correlation coefficient; *p*—level of statistical significance; ROM—range of movement; IPAQ—International Physical Activity Questionnaire; * statistical significance.

**Table 4:** Correlation of BMI and motor skills between groups.

Correlation of BMI with	CS (n = 32) r_s_ *p* *	MIS (n = 36) r_s_ *p* *
Walk for 50 m	0.39 0.027 *	0.35 0.03 *
ROM-hip flexion	−0.21 0.257	−0.37 0.020 *
ROM-hip abduction	−0.09 0.623	−0.23 0.170
Abductor muscle strength	−0.15 0.422	−0.24 0.151
IPAQ	−0.09 0.604	−0.27 0.111
Harris Hip Score	−0.06 0.733	−0.26 0.121

Legend: BMI—body mass index; CS—classical surgery; MIS—minimally invasive approach; r_s_—Spearman’s correlation coefficient; *p*—level of statistical significance; * statistical significance; ROM—range of movement; IPAQ—International Physical Activity Questionnaire.

**Table 5:** Correlation between age and motor skills between groups.

Correlation of Age with	CS (n = 32) r_s_ *p*	MIS (n = 36) r_s_ *p*
Harris Hip Score	−0.13 0.495	−0.36 0.032 *
Walk for 50 m	0.24 0.190	0.51 0.001 *
Abductor muscle strength	−0.14 0.451	−0.44 0.007 *

Legend: CS—classical surgery; MIS—minimally invasive surgery; r_s_—Spearman’s correlation coefficient; *p*—level of statistical significance; * statistical significance.

## Data Availability

The data generated by this research can be obtained from the corresponding author upon reasonable request.

## References

[1] Tudor A., Ruzic L., Vuckovic M., Prpic T., Rakovac I., Madjarevic T., Legovic D., Santic V., Mihelic R., Sestan B. (2016). Functional Recovery after Muscle Sparing Total Hip Arthroplasty in Comparison to Classic Lateral Approach—A Three Years Follow-up Study. J. Orthop. Sci. Off. J. Jpn. Orthop. Assoc..

[2] Roth A. (2012). [The minimally invasive anterolateral approach. A review of the literature]. Orthopade.

[3] Yang C., Zhu Q., Han Y., Zhu J., Wang H., Cong R., Zhang D. (2010). Minimally-Invasive Total Hip Arthroplasty Will Improve Early Postoperative Outcomes: A Prospective, Randomized, Controlled Trial. Ir. J. Med. Sci..

[4] Berry D.J., Berger R.A., Callaghan J.J., Dorr L.D., Duwelius P.J., Hartzband M.A., Lieberman J.R., Mears D.C. (2003). Minimally Invasive Total Hip Arthroplasty. Development, Early Results, and a Critical Analysis. Presented at the Annual Meeting of the American Orthopaedic Association, Charleston, South Carolina, USA, June 14, 2003. J. Bone Jt. Surg. Am..

[5] Woolson S.T., Mow C.S., Syquia J.F., Lannin J.V., Schurman D.J. (2004). Comparison of Primary Total Hip Replacements Performed with a Standard Incision or a Mini-Incision. J. Bone Jt. Surg. Am..

[6] Bertin K.C., Röttinger H. (2004). Anterolateral Mini-Incision Hip Replacement Surgery: A Modified Watson-Jones Approach. Clin. Orthop..

[7] Dorr L.D., Maheshwari A.V., Long W.T., Wan Z., Sirianni L.E. (2007). Early Pain Relief and Function after Posterior Minimally Invasive and Conventional Total Hip Arthroplasty. A Prospective, Randomized, Blinded Study. J. Bone Jt. Surg. Am..

[8] Mow C.S., Woolson S.T., Ngarmukos S.G., Park E.H., Lorenz H.P. (2005). Comparison of Scars from Total Hip Replacements Done with a Standard or a Mini-Incision. Clin. Orthop..

[9] Ritter M.A., Meding J.B. (1987). Total Hip Arthroplasty. Can the Patient Play Sports Again?. Orthopedics.

[10] Russo M.W., Macdonell J.R., Paulus M.C., Keller J.M., Zawadsky M.W. (2015). Increased Complications in Obese Patients Undergoing Direct Anterior Total Hip Arthroplasty. J. Arthroplast..

[11] Foote J., Panchoo K., Blair P., Bannister G. (2009). Length of Stay Following Primary Total Hip Replacement. Ann. R. Coll. Surg. Engl..

[12] Inneh I.A., Iorio R., Slover J.D., Bosco J.A. (2015). Role of Sociodemographic, Co-Morbid and Intraoperative Factors in Length of Stay Following Primary Total Hip Arthroplasty. J. Arthroplast..

[13] Liu W., Wahafu T., Cheng M., Cheng T., Zhang Y., Zhang X. (2015). The Influence of Obesity on Primary Total Hip Arthroplasty Outcomes: A Meta-Analysis of Prospective Cohort Studies. Orthop. Traumatol. Surg. Res. OTSR.

[14] Barrett M., Prasad A., Boyce L., Dawson-Bowling S., Achan P., Millington S., Hanna S.A. (2018). Total Hip Arthroplasty Outcomes in Morbidly Obese Patients: A Systematic Review. EFORT Open Rev..

[15] Haynes J., Nam D., Barrack R.L. (2017). Obesity in Total Hip Arthroplasty: Does It Make a Difference?. Bone Jt. J..

[16] Sayed-Noor A.S., Mukka S., Mohaddes M., Kärrholm J., Rolfson O. (2019). Body Mass Index Is Associated with Risk of Reoperation and Revision after Primary Total Hip Arthroplasty: A Study of the Swedish Hip Arthroplasty Register Including 83,146 Patients. Acta Orthop..

[17] Shen J., Chen D. (2014). Recent Progress in Osteoarthritis Research. J. Am. Acad. Orthop. Surg..

[18] Perruccio A.V., Young J.J., Wilfong J.M., Power J.D., Canizares M., Badley E.M. (2024). Osteoarthritis Year in Review 2023: Epidemiology & Therapy. Osteoarthr. Cartil..

[19] Rosenlund S., Broeng L., Holsgaard-Larsen A., Jensen C., Overgaard S. (2017). Patient-Reported Outcome after Total Hip Arthroplasty: Comparison between Lateral and Posterior Approach. Acta Orthop..

[20] Liljensøe A., Laursen J.O., Søballe K., Mechlenburg I. (2019). Is High Body Mass Index a Potential Risk Factor for Poor Outcome after Hip Arthroplasty? A Cohort Study of 98 Patients 1 Year after Surgery. Acta Orthop. Belg..

[21] D’Arrigo C., Speranza A., Monaco E., Carcangiu A., Ferretti A. (2009). Learning Curve in Tissue Sparing Total Hip Replacement: Comparison between Different Approaches. J. Orthop. Traumatol. Off. J. Ital. Soc. Orthop. Traumatol..

[22] Leuchte S., Luchs A., Wohlrab D. (2007). [Measurement of ground reaction forces after total hip arthroplasty using different surgical approaches]. Z. Orthop. Ihre Grenzgeb..

[23] Inaba Y., Kobayashi N., Yukizawa Y., Ishida T., Iwamoto N., Saito T. (2011). Little Clinical Advantage of Modified Watson-Jones Approach over Modified Mini-Incision Direct Lateral Approach in Primary Total Hip Arthroplasty. J. Arthroplast..

[24] Godoy-Monzon D., Buttaro M., Comba F., Piccaluga F., Cid-Casteulani A., Ordas A. (2019). Comparative Study of Radiological and Functional Outcomes Following a Direct Anterior Approach versus to a Posterolateral Approach to the Hip. Rev. Esp. Cir. Ortop. Traumatol..

[25] Jianbo J., Ying J., Xinxin L., Lianghao W., Baoqing Y., Rongguang A. (2019). Hip Hemiarthroplasty for Senile Femoral Neck Fractures: Minimally Invasive SuperPath Approach versus Traditional Posterior Approach. Injury.

[26] STROBE. https://www.strobe-statement.org/.

[27] Deng H.B., Macfarlane D.J., Thomas G.N., Lao X.Q., Jiang C.Q., Cheng K.K., Lam T.H. (2008). Reliability and Validity of the IPAQ-Chinese: The Guangzhou Biobank Cohort Study. Med. Sci. Sports Exerc..

[28] Macfarlane D.J., Lee C.C.Y., Ho E.Y.K., Chan K.L., Chan D.T.S. (2007). Reliability and Validity of the Chinese Version of IPAQ (Short, Last 7 Days). J. Sci. Med. Sport.

[29] Vandelanotte C., Bourdeaudhuij I., Philippaerts R., Sjostrom M., Sallis J. (2005). Reliability and Validity of a Computerized and Dutch Version of the International Physical Activity Questionnaire (IPAQ). J. Phys. Act Health.

[30] Mišigoj-Duraković M. (2018). Tjelesno Vježbanje i Zdravlje.

[31] Singh J.A., Schleck C., Harmsen S., Lewallen D. (2016). Clinically Important Improvement Thresholds for Harris Hip Score and Its Ability to Predict Revision Risk after Primary Total Hip Arthroplasty. BMC Musculoskelet. Disord..

[32] Nilsdotter A., Bremander A. (2011). Measures of Hip Function and Symptoms: Harris Hip Score (HHS), Hip Disability and Osteoarthritis Outcome Score (HOOS), Oxford Hip Score (OHS), Lequesne Index of Severity for Osteoarthritis of the Hip (LISOH), and American Academy of Orthopedic Surgeons (AAOS) Hip and Knee Questionnaire. Arthritis Care Res..

[33] Harris Hip Score—Orthopaedic Scores. https://www.orthopaedicscore.com/scorepages/harris_hip_score.html.

[34] Heller G.Z., Manuguerra M., Chow R. (2016). How to Analyze the Visual Analogue Scale: Myths, Truths and Clinical Relevance. Scand. J. Pain.

[35] Lenhard W., Lenhard A. (2017). Computation of Effect Sizes.

[36] Srikanth V.K., Fryer J.L., Zhai G., Winzenberg T.M., Hosmer D., Jones G. (2005). A Meta-Analysis of Sex Differences Prevalence, Incidence and Severity of Osteoarthritis. Osteoarthr. Cartil..

[37] Hughes-Oliver C.N., Srinivasan D., Schmitt D., Queen R.M. (2018). Gender and Limb Differences in Temporal Gait Parameters and Gait Variability in Ankle Osteoarthritis. Gait Posture.

[38] Conaghan P.G. (2013). Osteoarthritis in 2012: Parallel Evolution of OA Phenotypes and Therapies. Nat. Rev. Rheumatol..

[39] Crawford R.W., Murray D.W. (1997). Total Hip Replacement: Indications for Surgery and Risk Factors for Failure. Ann. Rheum. Dis..

[40] von Rottkay E., Rackwitz L., Rudert M., Nöth U., Reichert J.C. (2018). Function and Activity after Minimally Invasive Total Hip Arthroplasty Compared to a Healthy Population. Int. Orthop..

[41] Judge A., Batra R.N., Thomas G.E., Beard D., Javaid M.K., Murray D.W., Dieppe P.A., Dreinhoefer K.E., Peter-Guenther K., Field R. (2014). Body Mass Index Is Not a Clinically Meaningful Predictor of Patient Reported Outcomes of Primary Hip Replacement Surgery: Prospective Cohort Study. Osteoarthr. Cartil..

[42] McCalden R.W., Charron K.D., MacDonald S.J., Bourne R.B., Naudie D.D. (2011). Does Morbid Obesity Affect the Outcome of Total Hip Replacement?: An Analysis of 3290 THRs. J. Bone Jt. Surg. Br..

[43] Abdulla I., Mahdavi S., Khong H., Gill R., Powell J., Johnston K.D., Sharma R. (2020). Does Body Mass Index Affect the Rate of Adverse Outcomes in Total Hip and Knee Arthroplasty? A Retrospective Review of a Total Joint Replacement Database. Can. J. Surg. J. Can. Chir..

[44] Mouchti S., Whitehouse M.R., Sayers A., Hunt L.P., MacGregor A., Blom A.W. (2018). The Association of Body Mass Index with Risk of Long-Term Revision and 90-Day Mortality Following Primary Total Hip Replacement: Findings from the National Joint Registry for England, Wales, Northern Ireland and the Isle of Man. J. Bone Jt. Surg. Am..

[45] Courtine M., Bourredjem A., Gouteron A., Fournel I., Bartolone P., Baulot E., Ornetti P., Martz P. (2023). Functional Recovery after Total Hip/Knee Replacement in Obese People: A Systematic Review. Ann. Phys. Rehabil. Med..

